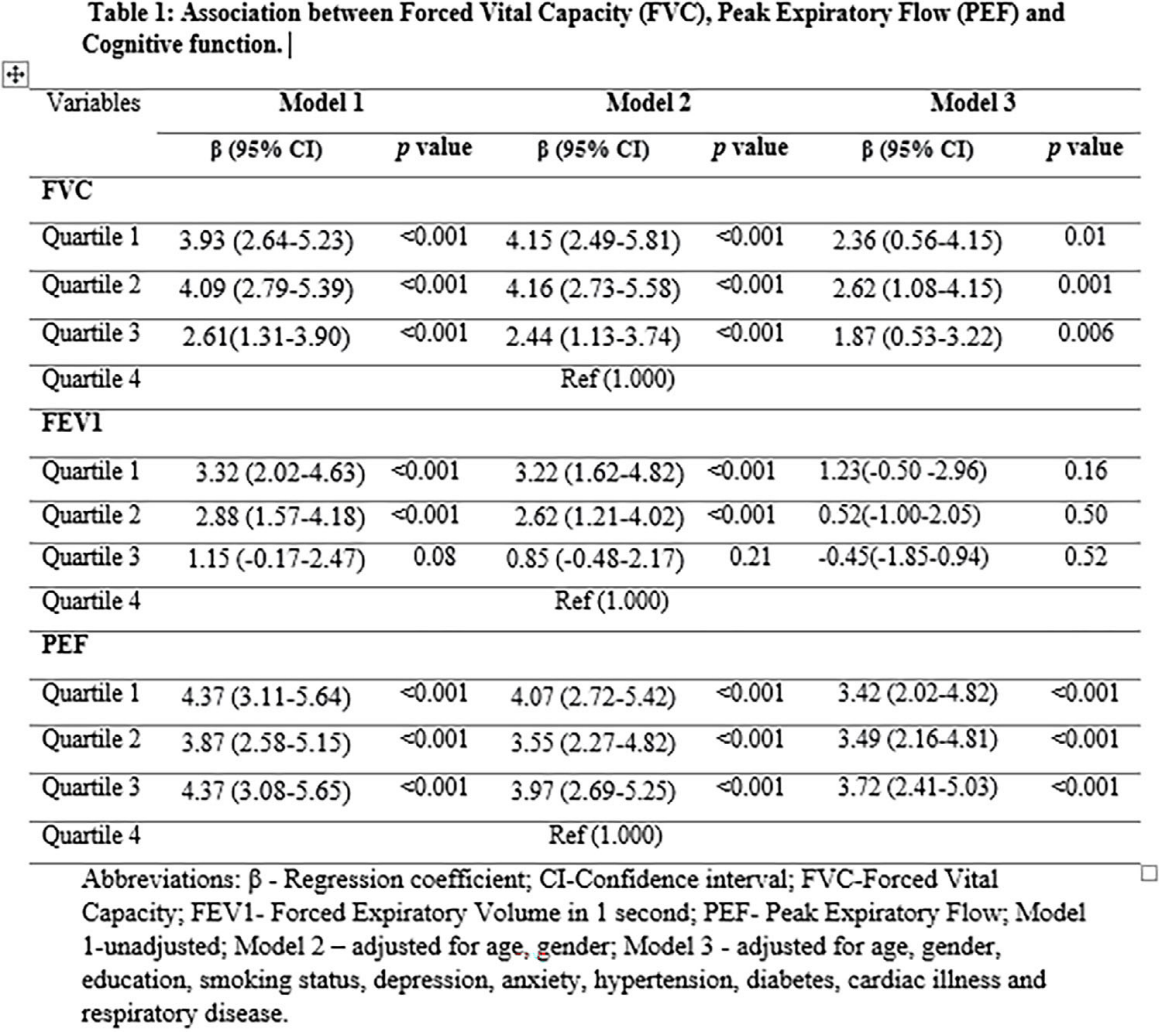# Association between pulmonary function and cognition in urban Indian adults – A cross sectional analysis

**DOI:** 10.1002/alz.091189

**Published:** 2025-01-03

**Authors:** Divya N Mallikarjun, Monisha S, Abhishek Mensegere Lingegodwa, Albert Stezin, Ajith Partha, Amitha C M, Rajitha Narayanasamy, Meghana R, Meenakshi Menon, Goutham Velavarajan, Vindhya Vishwanath, Palash K Malo, Sunitha HS, Deva Kumar HS, Deepashri Agrawal, Shafeeq K Shahul Hameed, Prathima Arvind, Jonas S. Sundarakumar, Thomas Gregor Issac

**Affiliations:** ^1^ Centre for Brain Research, Indian Institute of Science, Bangalore, Karnataka India

## Abstract

**Background:**

Pulmonary Function Tests (PFTs) are the non‐invasive tests to measure the lung function. Relationship between pulmonary function and cognition is an emerging area of research, understanding this is crucial for prevention and management of dementia. Hence this study aims to investigate the association between pulmonary function and cognition.

**Method:**

This cross sectional analysis includes the participants aged 45 years and above (N = 924) from an ongoing longitudinal study, the Tata Longitudinal Study of Aging (TLSA), conducted in urban Bangalore, India. The pulmonary function was assessed through spirometry, Z scores were calculated for Forced Vital Capacity (FVC), Forced Expiratory Volume in 1 second (FEV1), and Peak Expiratory Flow (PEF), then categorized into equal quartiles. Cognition was assessed using Addenbrooke’s Cognitive Examination III (ACE III). The association between pulmonary function and cognition was examined using multiple linear regression with three models: Model 1 – unadjusted, model 2‐adjusted for age and gender, model 3 – adjusted for age, gender, education, smoking status, depression, anxiety, hypertension, diabetes, cardiac illness and respiratory disease.

**Result:**

Regression analysis revealed an association between pulmonary function and cognition across all the models. In the fully adjusted model, compared to the lowest quartile (quartile 4), participants in highest quartiles of FVC performed better in ACE [quartile 1 (β **=** 2.36, 95% CI, 0.56‐4.15, *p* = 0.01), quartile 2 (β **=** 2.62, 95% CI, 1.08‐4.15, *p* = 0.001), quartile 3 (β **=** 1.87, 95% CI, 0.53‐3.22, *p* = 0.006)], similarly for PEF, participants in highest quartiles exhibited better ACE score [quartile 1 (β **=** 3.42, 95% CI, 2.02‐4.82, *p* <0.001), quartile 2 (β **=** 3.49, 95% CI, 2.16‐4.81, *p* <0.001), quartile 3 (β **=** 3.72, 95% CI, 2.41‐5.03, *p* ≤0.001)], there was no significant association found between FEV1 and ACE score.

**Conclusion:**

These findings indicate the link between pulmonary function and cognitive performance, highlighting the importance of respiratory well‐being in preserving the cognitive function.